# Study of Body Composition and Motor Skills of Futsal Athletes of Different Competitive Levels

**DOI:** 10.3390/sports12050137

**Published:** 2024-05-20

**Authors:** João Belo, João Valente-dos-Santos, João R. Pereira, Pedro Duarte-Mendes, José M. Gamonales, Rui Paulo

**Affiliations:** 1 Physical Education and Exercise and Health, Faculty of Physical Education and Sport, Lusófona University, 1749-024 Lisbon, Portugal; j.valente-dos-santos@hotmail.com (J.V.-d.-S.); p5916@ulusofona.pt (J.R.P.); 2 CIDEFES—Research Center for Sport, Physical Education and Exercise and Health, Faculty of Physical Education and Sport, Lusófona University, 1749-024 Lisbon, Portugal; 3 COD—Center of Sports Optimization, Sporting Clube de Portugal, 1600-464 Lisbon, Portugal; 4 Polytechnic Institute of Castelo Branco, 6000-084 Castelo Branco, Portugal; pedromendes@ipcb.pt (P.D.-M.); ruipaulo@ipcb.pt (R.P.); 5 Sport Physical Activity and Health Research & INnovation CenTer, SPRINT, 2001-904 Santarém, Portugal; 6 Sport, Health & Exercise Research Unit (SHERU), Polytechnic Institute of Castelo Branco, 6000-084 Castelo Branco, Portugal; 7 Training Optimization and Sports Performance Research Group (GOERD), Faulty of Sport Science, University of Extremadura, 10005 Cáceres, Spain; martinga-monales@unex.es; 8 Faculty of Health Sciences, Francisco de Vitoria University, 28223 Madrid, Spain; 9 Doctoral Program in Education and Technology, Distance University of Madrid, 28400 Madrid, Spain

**Keywords:** futsal, body composition, motor skills, competitive levels

## Abstract

This study aimed to verify whether there are differences in the body composition, functionality, lower-limb power, agility, and cardiorespiratory capacity in futsal players, comparing futsal athletes by competitive level. The athletes (N = 84) were divided into three groups: group Elite (N = 29), group Sub-Elite (N = 29), and group Non-Elite (N = 26). Anthropometric variables were analyzed through a bioimpedance scale (Inbody 270), and functionality was analyzed through a functional movement screen battery. The power of the lower limbs was tested with the Abalakov jump, the agility with the zigzag agility test, and the cardiorespiratory capacity through the futsal intermittent endurance test. Anthropometric data from futsal athletes revealed a homogeneity in relation to the variables analyzed, regardless of the level of competition in which they operate. In performance variables, the power of the members and functionality was considered a discriminating factor of the level of competitiveness of the athletes, with the Elite group athletes presenting the best values. We concluded that there were no differences in relation to the body composition of the athletes. However, the athletes of higher levels, as a rule, present better performances in physiological aspects, results that can be explained by the fact that there is a better periodization in terms of training, with more intense loads and more complex competitive calendars, thus resulting in a greater specialization of these athletes.

## 1. Introduction

Futsal is an indoor sport played by five players on each team and ruled internationally by FIFA; according to this organization, there are approximately 12 million players worldwide. Despite being a more dynamic sport, it is considered a variant of football due to its greater intensity [1], requiring athletes to quickly adapt their actions during the game to their team and opponents in less time and space [2]. This sport requires movement in various directions, as well as accelerations, decelerations, and intense direction changes, with an average of around 670 of these actions being observed during the match [3].

These high-intensity actions are interspersed with brief recovery periods, making this an intermittent sport [4,5,6], which leads to high-intensity efforts. Due to these short, high-intensity interval efforts and the total match duration (two halves of 20 min timed), futsal players need to use both aerobic and anaerobic energy systems [7]. To the author’s knowledge, no studies associate lower-limb strength/power with futsal performance. Nevertheless, Ref. [8] indicates that maximum strength and lower-limb power are essential for increasing performance. Therefore, assuming that there are no data on this matter, the maximal strength and power of the lower limbs might improve futsal performance, mainly in kicking the ball, but also in sprinting, acceleration, decelerations, and change of direction. Taking these last three actions and since, agility is the physical ability to perform a rapid movement, changing speed or direction in response, or not, to a stimulus. More specifically, according to Sekulic et al. [9], agility can be divided into two different fractions: pre-planned and unplanned or reactive. The first is linked to the ability to change direction at speed. The second, unplanned or reactive agility, as the name suggests, is when an athlete performs a particular movement in response to a stimulus. For Little and Williams [10], athletes with greater ability to perform these behaviors at high speed will perform better in sports.

Finally, concerning functionality, unlike many studies that use the functional movement screen test battery to predict whether an athlete is more exposed to a higher risk of possible injury, Kyle Kiesel, Plisky, and Voight [11] and Lisman, O’Connor, Deuster, and Knapik [12], in ours and in the same way as some of the other studies carried out [13,14,15], we intend to check whether there are differences between competitive levels in terms of the quality of their fundamental movement patterns.

With regard to body composition, this one has gained prominence as a factor that hinders or facilitates better performance [16]. Thus, having favorable values as indicators, such as muscle mass, fat mass, and % body fat (BF), is extremely important for playing sports at a higher competitive level [17,18]. With regard to this last parameter, an athlete with a high %CG may have a lower sporting performance [19], more precisely in terms of aerobic power, maximum anaerobic power, and muscular endurance [20]. In futsal, Matzenbacher, Pasquarelli, Rabelo, and Stanganelli [21] state that the ideal %FGC for optimum performance is between 8% and 12%.

In our opinion, this study is of scientific importance, as it seeks to find signs that will help to understand the importance that these five variables can have on the performance and professional development of each athlete. Thus, it tries to understand whether the characteristics assessed affect the competitive level of athletes in this sport. Therefore, the present study aims to see if there are differences in body composition, functionality, lower-limb power, cardiorespiratory fitness, and agility in futsal players and compares competitive levels, with the hypothesis that there are differences in the variables assessed when comparing the competitive level of the athletes, with athletes from the Placard League (Elite) showing the most favorable results in each of the study variables.

## 2. Materials and Methods

The present research has a cross-sectional design, and the quantitative method was used to interpret the data.

### 2.1. Participants

Athletes from various teams and competitive levels took part in this study: two teams from the 1st National Division (Liga Placard), two teams from the 2nd National Division, and two teams from the District Championship, making up a total of 84 athletes (N = 84) aged between 17 and 39 (26.03 ± 5.57 years). These subjects were divided into three groups according to their competitive level. After selecting the teams and players, we defined the following exclusion criteria: players not registered with the Portuguese Football Federation and players who refused to take part or whom the coach excluded for technical reasons. It should be noted that not all the players took part in all the evaluations due to the effort management of each club’s coaching staff. A non-probabilistic, intentional, and convenience sample was used to collect our target population, and the sample was selected according to the objectives of the study.

### 2.2. Assessment Protocols

#### 2.2.1. Body-Composition Assessment

**Figure 1:**
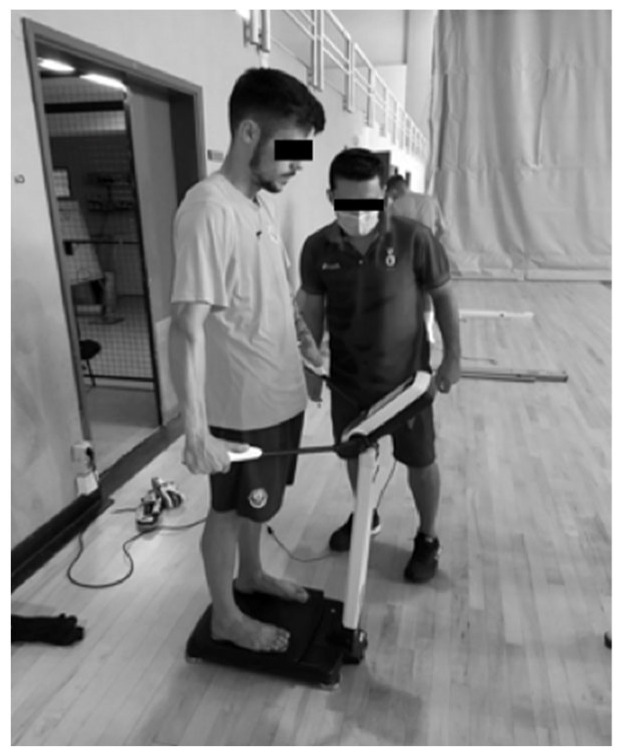
Assessment of an athlete’s body composition using the Inbody 270 bioimpedance scale.

The body-composition assessment was carried out using an Inbody 270 bioimpedance scale with an 8-electrode tetrapolar electrode system and frequencies of 20 and 100 kHz (Figure 1), making it possible to obtain values of skeletal muscle mass, fat mass, %BF, and body mass index (BMI). The reliability between the body-composition items evaluated was analyzed using Cronbach’s Alpha (0.797). We can consider that the assessments and instruments used are valid and reliable, according to the protocol of the study by Miller, Chambers, and Burns [22]. A portable stadiometer was used to enter the height value on the scale before they got on the scale.

#### 2.2.2. Functional Movement Screen (FMS)

**Figure 2:**
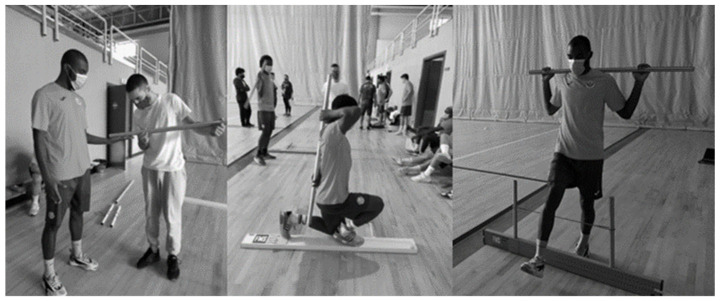
Functionality assessment using the FMS.

To assess the functionality of the subjects’ movement patterns, we used the FMS test battery (Figure 2); The entire implementation process was based on the Cook, Burton, Kisel, Rose, and Bryant guidelines [23], as well as the evaluation protocol and scoring system. Briefly, this screening system consists of seven tests of fundamental movements that require motor control, balance, mobility, and stability that follow the sequence: deep squat, hurdle step (left and right), in-line lunge (left and right), shoulder mobility (left and right), active straight leg raise (left and right), trunk stability push-up, and rotary stability (left and right). The reliability between the functionality items evaluated was analyzed using Cronbach’s Alpha (0.679). We can consider that the assessments and instruments used are valid and reliable according to the protocol of the guidelines by Cook et al. [23].

#### 2.2.3. Abalakov Jump (ABK)

**Figure 3:**
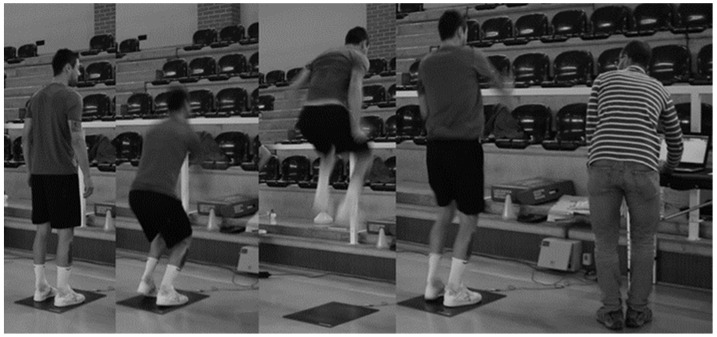
Elite athlete performing ABK test.

The ABK jump was used to assess the lower-limb power. This jump is similar to the countermovement jump. However, the upper limbs are free (hands do not need to stay at the hip), which can improve the jump height. Each player performed 3 ABK jumps; the best trial was recorded for analysis. To obtain the performance values for each athlete, we used the ChronoJump Boscosystem platform (Chronojump Boscosystem^®^, Barcelona, Spain). Using this tool, we collected lower-limb power values through each athlete’s vertical jumps (Figure 3), where we obtained the maximum height obtained in the best of the three jumps made. The reliability between the 3 jumps/repetitions performed was analyzed using Cronbach’s Alpha (0.853). We can consider that the assessments and the instruments used are valid and reliable, according to the protocol proposed by Bosco, Luhtanen, and Komi [24] and Markovic, Dizdar, Jukic, and Cardinale [25].

#### 2.2.4. Zig-Zag Agility Test

**Figure 4:**
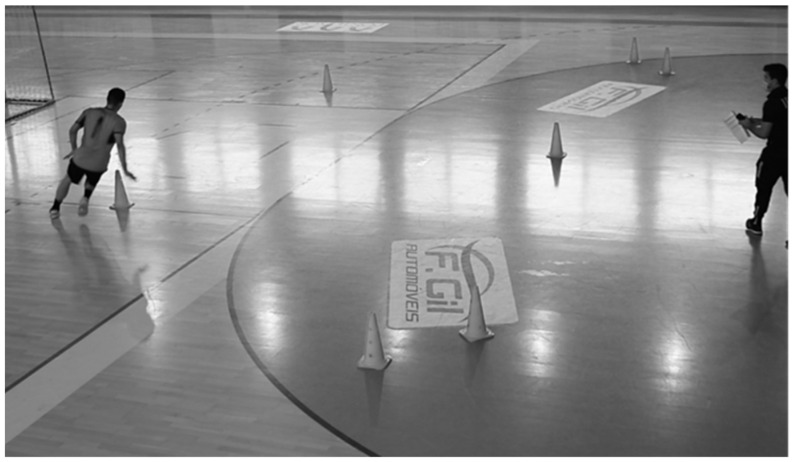
Evaluation of agility using the zig-zag agility test.

The zig-zag agility test was used to assess the ability to accelerate, decelerate, and change direction. Each player made 2 attempts; the best trial was recorded for analysis. This test consists of a 20 m course, where every five meters the athletes have to rotate at an angle of 100° (Figure 4). The reliability between the 2 attempts performed was analyzed using Cronbach’s Alpha (0.913). We can consider that the assessments and instruments used are valid and reliable according protocol proposed by Little and Williams [10].

#### 2.2.5. Futsal Intermittent Endurance Test (FIET)

**Figure 5:**
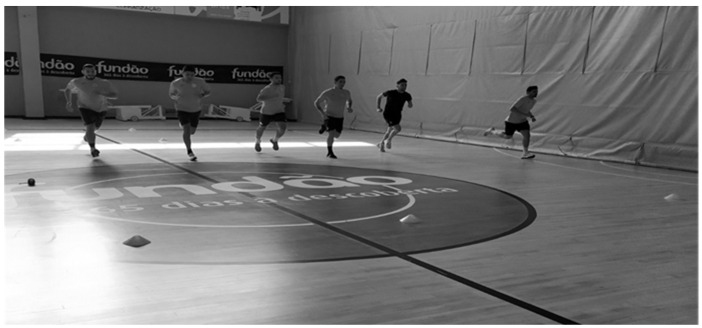
Assessment of cardiorespiratory capacity using FIET.

The FIET was used to assess cardiorespiratory fitness. This was developed by Barbero Alvarez et al. [26] to test futsal players’ ability to perform high-intensity intermittent exercise (Figure 5). According to Castagna and Barbero-Alvarez [27], the FIET consists of 45 m (3 × 15 m) shuttle runs performed at progressive speeds until exhaustion following an audio file. Every 45 min, the athletes have a 10 s rest period. After every 8 × 45 m, athletes rest for 30 s before continuing. The initial speed is set at 9 km/h, and the speed increments during the first 9 × 45 m episodes are 0.33 km/h1, successively changing to 0.20 km/h1 every 45 m until exhaustion. The test ends when the players fail to pass the control line (12 m) in time with beeps two times in succession. Similar to the study by Barbero-Alvarez et al. [28], the performance of each athlete was measured by the distance covered until exhaustion.

### 2.3. Procedures

Data was collected over a period of 2 months. This long collection time was largely due to the fact that it was carried out during the COVID-19 pandemic, which meant that we had to go to the clubs more than once in order to fully collect the data, as we only had a short time to contact the athletes each time (about an hour and a half each time). All the assessments were carried out at the facilities of the respective clubs and were divided into three different “stations”. At the first station, body composition (Figure 1) and vertical jumps (Figure 3) were assessed. At the second station, the functionality of the movement was evaluated using the FMS (Figure 2), and at the third station, the FIET test (Figure 5) and the agility test (Figure 4) were carried out. At the first and second stations, subjects were assessed in groups of two, while at the third station, they were assessed in groups of five, with at least two specialized and certified researchers always present. All the data collected from the six teams assessed was collected by the same research team using a record sheet.

It should be noted that all ethical procedures were taken into account, however, due to the characteristics of the research, the ethical approval in this manuscript was waived by the University’s Ethics Committee. Thus, each athlete trained for the assessments was given an anamnesis form and an informed consent form, and all ethical principles and international norms and standards relating to the Declaration of Helsinki and the Convention on Human Rights and Biomedicine [29] were respected and preserved.

### 2.4. Statistics Analysis

Data analysis was performed using the Statistical Package for the Social Sciences v.29.0 (SPSS) (IBM, Chicago, IL, USA). All collected data were grouped, and after an evaluation and the identification of discrepant values (outliers), these were excluded in order to minimize possible distortions of the results. The reliability between the items of each variable assessed and between the attempts carried out was analyzed using Cronbach’s Alpha.

Descriptive statistics were used to calculate means, standard deviation, and minimum and maximum values. A regression analysis was also carried out, taking into account the requirements: minimum number, independent residuals, absence of multicollinearity, absence of outliers, normally distributed residuals, homoscedasticity, and linear relationship between variables.

Using SPSS, the normality of the data distribution was verified. For the variables with a non-normal distribution, the Krushkal–Wallis test (Bonferroni correction) was used. For the remaining variables with normal distribution, the Anova, with a significance level set to alpha < 0.05 and alpha < 0.01, was performed. The inference method, based on the magnitude of the effects [30], was also analyzed.

## 3. Results

Regarding the regression analysis, the aim was to verify whether the body-composition variables and performance variables (functionality, agility, lower-limb power, and cardiorespiratory fitness) are capable of predicting the competitive level of athletes.

The regression analysis resulted in a statistically unacceptable model for the variables body mass, BMI, fat mass, and agility. On the other hand, the regression analysis resulted in a statistically accepted model for the variables: height [F (1,76) = 6.489; *p* = 0.013; R^2^ = 0.079]; fat-free mass [F (1.76) = 4.964; *p* = 0.029; R^2^ = 0.061]; body fat [F (1.76) = 4.574; *p* = 0.036; R^2^ = 0.057]; functionality [F (1,76) = 23.872; *p* < 0.001; R^2^ = 0.239]; lower-limb power [F (1.71) = 15.021; *p* < 0.001; R^2^ = 0.175]; and cardiorespiratory fitness [F (1,70) = 13.874; *p* < 0.001; R^2^ = 0.165], indicating that these variables could be predictors of the competitive level.

**Figure 6:**
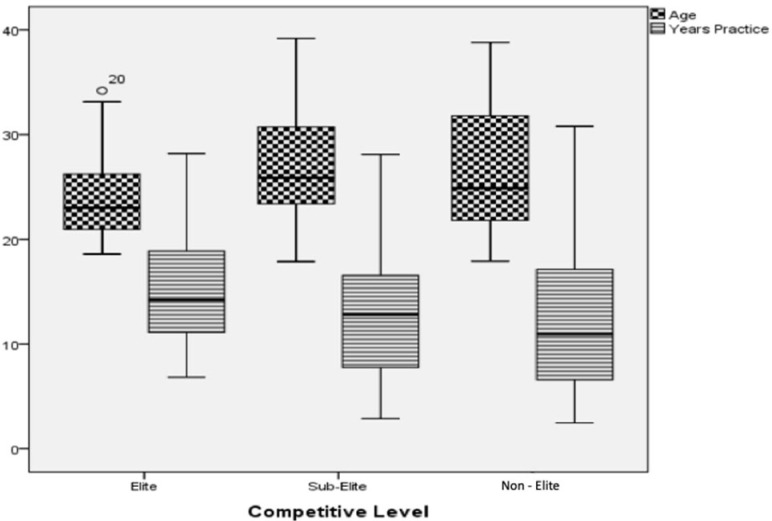
Group comparisons regarding the variables of age and years of practice.

In the present sample (Figure 6), the Elite group was younger than the Sub-Elite group (effect size = 0.62; *p* = 0.028).

Regarding height, it is possible to verify that the Non-Elite group is, on average, shorter than the Elite group (d = 0.71; *p* = 0.038) and the Sub-Elite group (d = 0.93; *p* = 0.002). Regarding body-composition measures, no significant differences were found between competitive levels, except for muscle mass. Players from the Sub-Elite group presented more muscle mass than the Non-Elite group (d = 0.64; *p* = 0.046), as shown in Table 1.

Table 2 displays differences between competitive groups according to the performance variables. The Elite group obtained significantly higher results on the ABK test compared to the Sub-Elite group (d = 1.20; *p* < 0.01) and the Non-Elite group (d = 1.17; *p* < 0.01). According to cardiorespiratory fitness, the Elite group, on average, covered a greater distance than the Non-Elite group (d = 1.01; *p* = 0.002). 

Lastly, the results of the FMS battery showed significant differences between the three groups. The Elite group obtained better results compared to the Sub-Elite group (d = 0.90; *p* = 0.004) and the Non-Elite group (d = 1.35; *p* < 0.01).

## 4. Discussion

The present study investigated age differences, years of practice, body composition, lower-limb power, agility, cardiorespiratory fitness, and functionality among futsal players according to their competitive levels.

There were significant differences between the Elite group and the Sub-Elite group. Players in the Elite group were the youngest, and those in the Sub-Elite group were the oldest. The study by Barbero Álvarez et al. [7] also found that players in the group with the highest competitive level (Professionals) were the youngest. The study by Naser and Ali [31] found that players from the lowest competitive level (Social Players) were the oldest, and those from the Semi-Elite group were the youngest. One of the factors that might explain these results is the great deal of investment in futsal training over the last decade. This has led players who have gone through this process to achieve higher performance levels and be better prepared to compete at higher competitive levels. Although players from the Elite Group were younger, elite players had more years of practice in the sport. Furthermore, players at higher competitive levels tend to compete at lower competitive levels as they age.

According to body composition, it is possible to highlight the variable height. Players from the Elite and Sub-Elite groups were significantly taller than the Non-Elite group. Previous studies [4,32] reported different findings, as no significant differences in height were reported. Body-composition indicators are discriminating factors regarding national team call-ups [18]. Therefore, it was hypothesized that taller players have more potential of being selected for national teams, particularly in certain positions as pivots or goalkeepers (GK). Consequently, future studies could combine not only differences by competitive level but also by playing position.

Another body-composition variable analyzed is BMI. According to the present sample, no differences were found between competitive levels. These results are aligned with previous studies [4,33]. Independently of the competitive level, more significance is given to the importance of body composition on players’ performance. Consequently, this might explain why no differences were found for BMI according to different competitive groups. Nevertheless, it has been said that [34] there is not enough evidence to say that indicators such as body mass are decisive for players at different competitive levels. Differences in fat-free mass were only verified between the Sub-Elite and Non-Elite groups; the Sub-Elite group had a higher fat-free mass.

Similarly, the study by Micheli et al. [35] shows that the higher the level at which an athlete competes, the higher their muscle mass. Also, the results of the study by Matias et al. [36], with players from the first, second, and third futsal divisions in Portugal, show that elite players have the highest average muscle-mass values. However, in this study, unlike ours, players from the second division have the lowest values. Despite these results, no significant differences were found when comparing the different competitive levels. These results could be due to the greater volume, intensity, and demands of training and competition. 

No differences were found between competitive levels for fat mass or %BF. Although there were no significant differences, we can see there is a tendency that, the higher the competitive level, the lower the body-fat value. Similarly, the results of the study by Sekulic et al. [37] also show that players at a higher competitive level tend to have lower %BF values, although there are no considerable differences. In the opposite direction are the results of the research by Matias et al. [36], where we can see that the lower the competitive level, the lower the fat mass and %BF the players have, with considerable differences between all the groups assessed.

This trend could be because players at higher competitive levels have better nutritional monitoring, since sports nutrition is essential for optimizing training and, consequently, sports competition. Bytomski [38] adds that there are also greater competitive and training demands.

Four indicators were considered to discriminate performance by competitive levels (low limb power, agility, functionality, and cardiorespiratory fitness). With regard to lower-limb power, elite players were significantly stronger than sub-elite or non-elite players. These results are in line with previous studies, both in futsal [39], where 33 players competing in the main Futsal league in Spain (LNFS) from two professional teams and one amateur team were assessed, and also in soccer [40], where, similar to this study, 81 players from different competitive levels (elite, sub-elite and non-elite) were compared. Nevertheless, it was previously reported [31] that lower-limb power did not differ between competitive levels. The fact that, in the present study, the Elite group was made up of players from the First National Division, most of whom were professionals, and this could be due to some of the factors already mentioned, such as more frequent weekly training sessions, higher quality and more demanding training, additional work in the gym, among others. However, this ability may not be as important for futsal as it is for other sports [34].

Contrary to the hypothesis proposed in this study, as far as the agility variable is concerned, no differences were found between the three groups. This converges with the results of the study by Sekulic et al. [41], where we found that lower-level players obtained better results in the agility test, the ability to change direction at speed without heading the ball, compared to higher-level players. Despite this result, it should be noted that tests were also carried out in the same study for reactive agility with and without driving the ball. In these tests, the players at a higher competitive level obtained better results. More recently, we can see different results in the study by Sekulic et al. [37], where players at a higher competitive level have higher average values when assessed in this capacity.

The evaluations were carried out on different types of surfaces (synthetic or wooden). So, the grip on the ground may have influenced the results, as the different characteristics of the surface influence the ability to change direction during futsal practice [42]. Another possible reason for these results could be that photoelectric cells were not used, which could have influenced the accuracy of the data collected.

Cardiorespiratory fitness was assessed using the FIET test [26]. In this test, elite players traveled more meters than sub-elite or non-elite players. These results are consistent with previous studies [7,31], which reported better cardiorespiratory fitness in elite groups than in lower levels. Therefore, it is plausible that this physiological capacity can be a determining factor in an athlete’s ability to reach higher competitive levels [34]. According to Álvarez et al. [7], players at higher competitive levels have a training load three times greater than the lower-ranking players and, therefore, have more chances to improve this physiological capacity.

On the other hand, elite divisions/leagues in which elite players participate are extremely demanding competitions. Therefore, elite professional players might improve cardiorespiratory fitness not only by better training planning, better training conditions, and more training volume but also by more frequent, intense, and demanding competitions. This factor will inevitably increase the gap between the competitive-level groups even more.

Lastly, functionality was also assessed in the players’ performance section. The FMS test was the only one where the higher-level group was better than the lower-level group, independently of the competitive levels (i.e., elite > sub-elite; elite > non-elite; and sub-elite > non-elite). To the best of the authors’ knowledge, no studies associate FMS results with the competitive levels of futsal players. However, it seems that players of higher competitive levels of American football [43] and golf [44] achieved better results on the FMS test when compared with lower competitive athletes of the same sport.

These differences may be because players at higher competitive levels have more and better support in terms of complementary training focusing on muscle strengthening/balance and proprioceptive work since special attention is now being paid to this type of work, as several studies are showing positive effects on functionality [45,46,47].

Our research was carried out during the acute phase of the pandemic, so we had some difficulties accessing the sample. The time available was very limited, meaning our research team had to go to each club several times. Even so, we included elite athletes from one of the best championships in the world in our sample. The scarcity of research on this specific topic also made the design of this study more complex since the vast majority of research focuses on comparing youth and senior players.

It should be noted that this study has shown that futsal players are homogeneous in terms of their body composition, regardless of the competitive level at which they play. Lower-limb power and functionality were considered to be discriminating factors in terms of the players’ competitive level, and the athletes in the Elite group were the ones with the best values in the vertical jump.

## 5. Conclusions

When we compared the competitive levels, we concluded that there were no differences in the players’ body compositions, and the hypothesis we had proposed was not verified. However, as expected, players at higher levels generally perform better in physiological aspects. These results might be explained by better training schedules with more intense loads and more complex competitive calendars, thus resulting in greater (professional) specialization of these players.

Since research into the causes that lead an athlete to reach a certain competitive level needs to be clarified, it is suggested that future research could relate players’ performance to their body composition. Since this sport has a significant technical and tactical component, future studies should also evaluate these two aspects.

## Figures and Tables

**Table 1:** Group comparisons regarding body-composition variables.

Dependent Variable	Competitive Level	N	Mean ± SD	Effect Size (d; ±95% CI)	Sig.
Height (cm)	Elite	27	173.85 ± 5.47	0.27 (−0.26 ± 0.8)	0.626
	Sub-Elite	28	175.46 ± 6.22	Small	
	Elite	27	173.85 ± 5.47	0.71 (0.16 ± 1.27)	0.038
	Non-Elite	26	169.42 ± 6.76	Moderate	
	Sub-Elite	28	175.46 ± 6.22	0.93 (0.36 ± 1.48)	0.002
	Non-Elite	26	169.42 ± 6.76	Moderate	
Body Mass (Kg)	Elite	27	74.06± 6.99	0,17 (−0.36 ± 0.70)	0.668
	Sub-Elite	28	75.63 ± 10.66	Trivial	
	Elite	27	74.06± 6.99	0.13 (−0.41 ± 0.67)	0.141
	Non-Elite	26	72.58 ± 14.57	Trivial	
	Sub-Elite	28	75.63 ± 10.66	0.24 (−0.30 ± 0.77)	0.095
	Non-Elite	26	72.58 ± 14.57	Small	
BMI (Kg/m^2^)	Elite	27	24.49 ± 1.86	0.02 (−0.51 ± 0.55)	0.528
	Sub-Elite	28	24.54 ± 3.16	Trivial	
	Elite	27	24.49 ± 1.86	0.22 (−0.32 ± 0.76)	0.711
	Non-Elite	26	25.19 ± 4.10	Small	
	Sub-Elite	28	24.54 ± 3.16	0.18 (−0.36 ± 0.71)	0.489
	Non-Elite	26	25.19 ± 4.10	Trivial	
Fat-free Mass (Kg)	Elite	27	35.35± 3.88	0.04 (−0.49 ± 0.57)	0.994
	Sub-Elite	28	35.51 ± 4.40	Trivial	
	Elite	27	35.35± 3.88	0.64 (0.09 ± 1.19)	0.062
	Non-Elite	26	32.46 ± 4.94	Moderate	
	Sub-Elite	28	35.51 ± 4.40	0.64 (0.10 ± 1.19)	0.046
	Non-Elite	26	32.46 ± 4.94	Moderate	
Fat Mass (Kg)	Elite	27	12.23 ± 3.71	0.18 (−0.35 ± 0.71)	0.926
	Sub-Elite	28	13.29 ± 7.12	Trivial	
	Elite	27	12.23 ± 3.71	0.49 (−0.06 ± 1.03)	0.348
	Non-Elite	26	15.43 ± 8.42	Small	
	Sub-Elite	28	13.29 ± 7.12	0.27 (0.26 ± 0.81)	0.213
	Non-Elite	26	15.43 ± 8.42	Small	
Body Fat (%)	Elite	27	16.48 ± 4.61	0.16 (−0.37 ± 0.69)	0.913
	Sub-Elite	28	17.52 ± 7.70	Trivial	
	Elite	27	16.48 ± 4.61	0.67 (0.11 ± 1.22)	0.067
	Non-Elite	26	20.49 ± 7.02	Moderate	
	Sub-Elite	28	17.52 ± 7.70	0.40 (−0.14 ± 0.94)	0.075
	Non-Elite	26	20.49 ± 7.02	Small	

**Table 2:** Group comparisons regarding the players’ performance variables in the ABK test, zig-zag agility test, FIET, and FMS.

Dependent Variable	Competitive Level	N	Mean ± SD	Effect Size (d; ±95% CI)	Sig.
ABK test—Highest Jump (cm)	Elite	21	45.50 ± 6.95	1.20 (0.59± 1.82)	<0.001
	Sub-Elite	28	37.89 ± 5.64	Moderate	
	Elite	21	45.50 ± 6.95	1.17 (0.55 ± −1.79)	<0.001
	Non-Elite	26	37.57 ± 6.42	Moderate	
	Sub-Elite	28	37.89 ± 5.64	0.05 (−0.48± 0.59)	0.983
	Non-Elite	26	37.57 ± 6.42	Trivial	
Zig Zag—shorter time (s)	Elite	23	5.71 ± 0.38	0.13 (−0.44± 0.69)	0.297
	Sub-Elite	26	5.66 ± 0.40	Trivial	
	Elite	23	5.71 ± 0.38	0.06 (−0.51± 0.63)	0.353
	Non-Elite	24	5.73 ± 0.28	Trivial	
	Sub-Elite	26	5.66 ± 0.40	0.20 (−0.36 ± 0.75)	0.168
	Non-Elite	24	5.73 ± 0.28	Trivial	
FIET— Distance traveled (m)	Elite	24	1113.75 ± 218.95	0.40 (−0.16± 0.96)	0.403
	Sub-Elite	26	1024.62 ± 222.76	Small	
	Elite	24	1113.75 ± 218.95	1.01 (0.42± 1.60)	0.002
	Non-Elite	26	870.58± 252.30	Moderate	
	Sub-Elite	26	1024.62 ± 222.76	0.64 (0.08 ± 1.19)	0.064
	Non-Elite	26	870.58± 252.30	Moderate	
FMS total Score	Elite	27	18.77 ± 1.18	0.90 (0.35± 1.46)	0.004
	Sub-Elite	28	17.64 ± 1.28	Moderate	
	Elite	27	18.77 ± 1.18	1.35 (0.75± 1.94)	<0.001
	Non-Elite	26	16.76 ± 1.72	High	
	Sub-Elite	28	17.64 ± 1.28	0.58 (0.03 ± 1.12)	0.039
	Non-Elite	26	16.76 ± 1.72	Small	

## Data Availability

The data is not publicly available due to ethical and privacy restrictions. The raw data, which support the conclusions of this article, will be made available by the authors upon request.
